# IL-15-secreting CAR natural killer cells directed toward the pan-cancer target CD70 eliminate both cancer cells and cancer-associated fibroblasts

**DOI:** 10.1186/s13045-024-01525-w

**Published:** 2024-02-09

**Authors:** Astrid Van den Eynde, Laura Gehrcken, Tias Verhezen, Ho Wa Lau, Christophe Hermans, Hilde Lambrechts, Tal Flieswasser, Delphine Quatannens, Gils Roex, Karen Zwaenepoel, Elly Marcq, Philippe Joye, Edgar Cardenas De La Hoz, Christophe Deben, Alessia Gasparini, Pierre Montay-Gruel, Maxim Le Compte, Eva Lion, Filip Lardon, Steven Van Laere, Vasiliki Siozopoulou, Diana Campillo-Davo, Jorrit De Waele, Patrick Pauwels, Julie Jacobs, Evelien Smits, Jonas R. M. Van Audenaerde

**Affiliations:** 1 Center for Oncological Research (CORE), Integrated Personalized and Precision Oncology Network (IPPON), Faculty of Medicine and Health Sciences, University of Antwerp, Wilrijk, Belgium; 2 Laboratory of Experimental Hematology (LEH), Vaccine and Infectious Disease Institute (VAXINFECTIO), Faculty of Medicine and Health Sciences, University of Antwerp, Edegem, Belgium; 3 Department of Pathology, Antwerp University Hospital, Edegem, Belgium; 4 Lab of Dendritic Cell Biology and Cancer Immunotherapy, VIB Center for Inflammation Research, Brussels, Belgium; 5 Brussels Center for Immunology, Vrije Universiteit Brussel, Brussels, Belgium; 6 Molecular Imaging Center Antwerp (MICA), University of Antwerp, Wilrijk, Belgium; 7 Industrial Vision Lab (InViLab), University of Antwerp, Antwerp, Belgium; 8 Iridium Netwerk, Radiation Oncology, Antwerp, Belgium; 9 Center for Cell Therapy and Regenerative Medicine (CCRG), Antwerp University Hospital, Edegem, Belgium; 10 University Hospital Saint-Luc, University of Louvain, Brussels, Belgium

## Abstract

**Background:**

It remains challenging to obtain positive outcomes with chimeric antigen receptor (CAR)-engineered cell therapies in solid malignancies, like colorectal cancer (CRC) and pancreatic ductal adenocarcinoma (PDAC). A major obstacle is the lack of targetable surface antigens that are not shared by healthy tissues. CD70 emerges as interesting target, due to its stringent expression pattern in healthy tissue and its apparent role in tumor progression in a considerable amount of malignancies. Moreover, CD70 is also expressed on cancer-associated fibroblasts (CAFs), another roadblock for treatment efficacy in CRC and PDAC. We explored the therapeutic potential of CD70 as target for CAR natural killer (NK) cell therapy in CRC, PDAC, focusing on tumor cells and CAFs, and lymphoma.

**Methods:**

RNA-seq data and immunohistochemical analysis of patient samples were used to explore CD70 expression in CRC and PDAC patients. In addition, CD70-targeting CAR NK cells were developed to assess cytotoxic activity against CD70^+^ tumor cells and CAFs, and the effect of cytokine stimulation on their efficacy was evaluated. The in vitro functionality of CD70-CAR NK cells was investigated against a panel of tumor and CAF cell lines with varying CD70 expression. Lymphoma-bearing mice were used to validate in vivo potency of CD70-CAR NK cells. Lastly, to consider patient variability, CD70-CAR NK cells were tested on patient-derived organoids containing CAFs.

**Results:**

In this study, we identified CD70 as a target for tumor cells and CAFs in CRC and PDAC patients. Functional evaluation of CD70-directed CAR NK cells indicated that IL-15 stimulation is essential to obtain effective elimination of CD70^+^ tumor cells and CAFs, and to improve tumor burden and survival of mice bearing CD70^+^ tumors. Mechanistically, IL-15 stimulation resulted in improved potency of CD70-CAR NK cells by upregulating CAR expression and increasing secretion of pro-inflammatory cytokines, in a mainly autocrine or intracellular manner.

**Conclusions:**

We disclose CD70 as an attractive target both in hematological and solid tumors. IL-15 armored CAR NK cells act as potent effectors to eliminate these CD70^+^ cells. They can target both tumor cells and CAFs in patients with CRC and PDAC, and potentially other desmoplastic solid tumors.

**Supplementary Information:**

The online version contains supplementary material available at 10.1186/s13045-024-01525-w.

## Introduction

The immune checkpoint molecule CD70 is increasingly being recognized as a pan-cancer target [1–3]. In healthy tissue, CD70 expression is restricted to the immune cell compartment where it acts through binding to its receptor CD27 as a co-stimulatory molecule regulating immune cell expansion and differentiation. To allow strictly coordinated execution of these complex mechanisms, CD70 is only transiently expressed upon activation on T cells, B cells, natural killer (NK) cells and mature dendritic cells [4, 5]. Yet, aberrant and constitutive expression of CD70 has been reported on both primary tumors and metastatic lesions of a wide array of hematological and solid tumor types [1]. Numerous studies have revealed that the dysregulation of the CD70-CD27 axis in tumor cells and microenvironment leads to immune evasion and tumor progression via different mechanisms [6, 7]. Consequently, the expression of CD70 on tumor cells has also been linked to worse prognosis in several malignancies [8–12].

In solid tumors, especially in pancreatic adenocarcinoma (PDAC), the intricate interactions between cancer cells and cancer-associated fibroblasts (CAFs) in the tumor microenvironment (TME) create a supportive niche for tumor growth and metastasis. Moreover, growing evidence indicates that CAFs play a key role in cancer immune evasion and drug resistance, making them an interesting target for therapy [13–15]. However, specific markers that can selectively differentiate tumor-promoting CAFs from tumor-restraining CAFs are needed [16, 17]. We and others have previously uncovered a subset of CD70-expressing CAFs in colorectal cancer (CRC) and head and neck squamous cell carcinoma, and identified them as an adverse prognostic factor for CRC patients [18–20]. Particularly in CRC, our study has demonstrated that these CD70^+^ CAFs promote tumor migration and regulate immune escape via accumulation of regulatory T cells [19]. Hence, CD70^+^ CAFs are a promising target to impede tumor migration and immune suppression in CRC and in potentially other tumor types like head and neck squamous cell carcinoma and PDAC.

Development of chimeric antigen receptor (CAR)-engineered cell therapies have revolutionized anti-cancer immunotherapies, however one of the major hurdles to translate the great successes of CAR T cell therapy as seen in hematological malignancies to solid tumors is the lack of good surface antigens, selective for tumor tissue [21]. Considering the specific expression on tumor cells and CAFs, CD70 emerges as an ideal target for CAR-based immunotherapy. In this regard, NK cells have gained a lot of interest as an alternative platform for CAR engineering, as CAR NK cells harbor many advantages over the extensively investigated CAR T cells. Apart from a more favorable treatment-related toxicity profile, CAR NK cells can be produced as an allogeneic off-the-shelf product, thus reducing production time and costs, and have intrinsic tumor killing capacity in addition to CAR-mediated cytotoxicity [22–24].

In this study, using RNA-seq data from the TCGA database and in-house immunohistochemical staining of tumor resections, we identified CD70-expressing tumor cells and CAFs as a promising therapeutic target in PDAC and CRC patients. Furthermore, we assessed if CD70^+^ tumor cells and CAFs can be eliminated by CD70-directed CAR NK cells using in vitro and in vivo models that are representative for the clinical situation of CD70^+^ tumors. Interestingly, CD70-CAR NK cells demonstrated to be highly competent in eliminating both CD70^+^ tumor cells and CAFs when stimulated with interleukin (IL)-15.

## Material and methods

### CD70 RNA-seq analysis

Pan-cancer bulk RNA-seq data from 28 different cancer types from the PCAWG Firehose cohort included in The Cancer Genome Atlas (TCGA) were downloaded from the cBioPortal for Cancer Genomics platform (https://www.cbioportal.org) using the package “cgdsr” in R (R Core Team) [25]. RNA-seq by Expectation–Maximization (RSEM) values were converted into integers, and genes with counts greater than 10 in at least 10% of the tumor samples per cancer type were filtered in for further analysis. Resulting expression profiles were scaled by the number of counts per million reads, log2-transformed, and quantile normalized. Single sample gene set enrichment analysis (ssGSEA–BioC-package “GSVA”) was performed on colon adenocarcinoma (COAD), rectal adenocarcinoma (READ) and pancreatic adenocarcinoma (PAAD) data sets for two published gene sets related to general CAFs and CAFs associated with resistance to immune checkpoint inhibition (ICB; [26]). Associations between CD70 expression and CAF (general CAFs and CAFs associated with ICB) abundance scores were tested using Spearman’s correlation coefficients or linear regression models using CD70 as the dependent variable. Comparison of different linear models was done using log ratio tests. In each case, p-values inferior to 0.05 were considered significant.

### Patient and healthy donor samples

Formalin-fixed paraffin embedded (FFPE) tissue blocks of 23 PDAC patients and nine historical PDAC FFPE sections previously involved in a clinical study [1] were provided by the Antwerp Biobank (Antwerp, Belgium; ID: BE71030031000) and approved by the Ethics Committee of the Antwerp University Hospital-University of Antwerp (UZA-UAntwerp) under study reference EC14/47/480 and EC13/47/469, respectively. Generation and usage of patient-derived PDAC organoids was approved by patients via written informed consent and by the UZA-UAntwerp Ethics Committee under study reference EC14/47/480. Buffy coats from four healthy donors were purchased from the Blood Transfusion Center of the Red Cross-Flanders (Mechelen, Belgium) and usage was approved by the UZA-UAntwerp Ethics Committee under study reference EC5488.

### Immunohistochemistry

CD70 expression in the TME of PDAC patients was evaluated on 23 tumor resections by manual staining for hematoxylin and eosin (H&E), alpha-smooth muscle actin (α-SMA), and CD70 on consecutive sections from FFPE tissue blocks. Five µm-thick sections were baked for 2 h at 60°C and exposed to heat-induced epitope retrieval by incubation in Envision FLEX+ antigen retrieval solution (DAKO; 20 min) at 97°C (PT-Link instrument, DAKO). Subsequently, endogenous peroxidase activity was quenched by incubating the slides in peroxidase blocking buffer (DAKO; 5 min). Immunohistochemical staining of CD70 using an anti-CD70 polyclonal antibody (#PA5-102557, 1:750, Thermo Fisher) was performed with the protocol previously described [27]. After blocking with normal goat serum (Jackson Immuno Research; 30 min), the anti-CD70 antibody was incubated at room temperature (35 min), followed by rabbit enhanced polymer-based linker incubation (DAKO; 15 min), and visualized using the Envision FLEX+ detection kit (DAKO; 25 min) according to the instructions of the manufacturer. Immunohistochemical staining of α-SMA using an anti-α-SMA monoclonal antibody (mAB; clone 1A4, 1:100, Agilent) was performed in an analogous manner to CD70 without blocking with normal goat serum or addition of a rabbit enhanced polymer-based linker. All sections were counterstained with hematoxylin (Merck; 2 min), dehydrated and mounted with Expert mounting medium (Cellpath). Tonsil and placenta tissues were used as positive controls for CD70 and α-SMA staining, respectively.

Scoring for CD70 positivity was restricted to the stromal compartment and performed by pathologists experienced in immunohistochemical staining patterns of CD70 [1, 19, 28, 29]. Positive staining was assigned when CAFs displayed strong membrane or cytoplasmic intensity and was expressed as a percentage of positive CAFs of the total stromal fibroblasts. To rule out non-specific binding of the CD70 antibody in the stromal compartment, nine historical PDAC tissue sections that were stained with a clinically approved anti-CD70 mAb (clone 301731, 1:40, R&D Systems, not commercially available anymore) were included [1].

Dissected tumors from Raji cell line-bearing NOD-*Prkdc*^*scid*^*-IL2rg*^*Tm1*^/Rj (NSG) mice were fixed in 4% formaldehyde, processed, and paraffin embedded. Five µm-thick sections from the FFPE tissue blocks were stained for H&E and CD70 as described above.

### Cell lines and culture conditions

The NK-92 and Raji cell lines were purchased from the German Collection of Microorganisms and Cell Cultures, the LIM2099 cell line from Sigma-Aldrich, and the PANC-1 cell line from the American Type Culture Collection. The CT5.3hTERT, RLT-PSC, and hPSC21 CAF cell lines were kindly provided by Prof. O. De Wever (Ghent University, Ghent, Belgium) [30], Prof. R. Jesenofsky (University of Heidelberg, Mannheim, Germany) [31], and Prof. A. Masamune (Tohoku University Graduate School of Medicine, Sendai, Miyagi Prefecture, Japan) [32], respectively.

NK-92 cells were cultured in GlutaMAX alpha Minimum Essential Medium (α-MEM; Life Technologies) supplemented with 12.5% Fetal Bovine Serum (FBS; Life Technologies), 12.5% horse serum (Life Technologies), 2mM L-glutamine (Life Technologies), 1% Penicillin/Streptomycin (P/S; Life Technologies) and 150 U/mL recombinant IL-2 (ImmunoTools), as described before [33]. Raji and LIM2099 cells were cultured in Roswell Park Memorial Institute medium (Life Technologies) supplemented with 10% FBS, 2mM L-glutamine, and 1% P/S. CT5.3hTERT and PANC-1 cells were cultivated in Dulbecco-Modified Eagle Medium (DMEM; Life Technologies) supplemented with 10% FBS, 2mM L-glutamine, and 1% P/S. Lastly, RLT-PSC, and hPSC21 cell lines were cultured in DMEM/F12 (Life Technologies) supplemented with 10% FBS, 2mM L-glutamine, and 1% P/S. Cell cultures were maintained in exponential growth in 5% CO_2_ in a humidified incubator at 37°C, confirmed Mycoplasma free through regular testing with the Mycoalert Mycoplasma detection kit (Lonza), and their identity validated by short tandem repeat profiling.

### Generation of CD70-CAR NK cells

CD70-CAR and CD70-CAR-IL-15 constructs were designed via Creative Biolabs using the CellRapeutics™ CAR Technology platform and inserted into a pST1 vector (kindly provided by Prof. Uğur Şahin, University Medical Center Mainz, Mainz, Germany) behind the T7 promoter site. The pST1-CD70-CAR and pST1-CD70-CAR-IL-15 plasmids were transformed in *Escherichia coli* (SoloPack Gold Supercompetent Cells, Agilent) using a heat shock of 42°C for 30 s, selected on Luria–Bertani broth agar (Miller; Sigma-Aldrich) plates containing Kanamycin (Sigma-Aldrich), amplified in Luria–Bertani broth medium (Miller; Sigma-Aldrich) containing Kanamycin, purified with the NucleoBond Xtra Midi Plus EF kit (Macherey Nagel), and linearized with the PmeI restriction enzyme (Life Technologies). In vitro transcription of CD70-CAR- and CD70-CAR-IL-15-encoding messenger RNA (mRNA) was performed using the mMESSAGE mMACHINE T7 transcription kit (Life Technologies) following manufacturer’s instructions. NK-92 cells were electroporated as described elsewhere [33]. In short, cells were pulsed using a Gene Pulser Xcell (Bio-Rad) with time constant protocol (300V, 12 ms) in 4 mm cuvette (ImmunoSource), dissolved in 200 μL Opti-MEM (Life Technologies) at a concentration of 25–125 × 10^6^ cells/mL in the presence of 100 μg/mL CD70-CAR- or CD70-CAR-IL-15-encoding mRNA. NK-92 cells electroporated without CAR-encoding mRNA (MOCK) were used as control cells. Electroporated cells were recovered in NK-92 medium without IL-2 for at least 4 h until further use in downstream applications. IL-2 was never supplemented in downstream experiments unless otherwise stated.

### Flow cytometric phenotyping

Expression of CD70 was assessed on peripheral blood mononuclear cells (PBMCs) from healthy donors by flow cytometry. PBMCs were isolated by Lymphoprep (Stemcell technologies) density gradient centrifugation from buffy coats of four healthy donors, purchased from the Blood Transfusion Center of the Red Cross-Flanders. Prior to antibody staining, PBMCs were incubated 30 min at 4°C with human serum (Sigma-Aldrich) to prevent aspecific binding to Fc receptors. Subsequently, PBMCs were stained for 30 min at 4°C with a multicolor panel containing CD3-FITC (clone SK7, Biolegend), CD4-BB700 (Clone SK3, Biolegend), CD8-BV570 (Clone RPA-T8, Biolegend), CD56-PE-Cy7 (NCAM16.2, BD Biosciences), CD19-BV421 (Clone HIB19, BD Biosciences), CD14-BV785 (Clone 63D3, Biolegend), and CD70-PE (Clone ki-24, BD Biosciences). Live-Dead Fixable Near-IR (Thermo Fisher) was included to exclude non-viable cells from the analysis. Acquisition was performed on a NovoCyte Quanteon (Agilent Technologies).

Surface expression of the CD70-CAR and CD70-CAR-IL-15 constructs on NK-92 cells was evaluated via flow cytometry by staining with a CD27-PE mAb (Clone O323, Cell Signaling Technology) or corresponding isotype control IgG1κ mAb (Clone MOPC-21, Cell Signaling Technology) for 30 min at 4°C. Expression of CD70 on tumor and CAF cell lines was determined by staining with a CD70-PE mAb or corresponding isotype control IgG3κ mAb (Clone J606, BD Biosciences) for 30 min at 4°C. CD27 and CD70 expression were measured on a CytoFLEX (Beckman Coulter) after 15 min incubation with the fluorescent intercalating viability dye 7-amino-actinomycin D (7-AAD; Biolegend) to exclude non-viable cells.

Expression of immune checkpoint molecules DNAM-1, TACTILE, TIGIT, PD-1, and LAG-3 was determined on MOCK, CD70-CAR, and CD70-CAR-IL-15 NK cells 24 h post-electroporation by pretreating the cells with human serum for 30 min at 4°C, staining the cells with a multicolor antibody panel for 30 min at 4°C, and measuring the samples using a NovoCyte Quanteon. The multicolor antibody panel included DNAM-1-FITC (Clone11A8, Biolegend), CD96-BV421 (Clone NK92.39, Biolegend), TIGIT-PE-Dazzle (Clone A15153G, Biolegend), PD-1-BV650 (Clone EH12.2H7, Biolegend), and LAG3-BV785 (Clone 11C3C65, Biolegend). Live-Dead Fixable Near-IR marker was used to exclude non-viable cells from the analysis.

### Analysis of IL-15, IFN-γ and TNF-α secretion

In order to analyze secretion of the IL-15, IFN-γ and TNF-α pro-inflammatory cytokines by CD70-CAR, CD70-CAR-IL-15, or MOCK control NK cells into the supernatant, cells were seeded after electroporation in flat-bottom 96-well plates (Greiner bio-one) at a concentration of 1 × 10^6^ cells/mL. Supernatant was taken 4 h, 24 h, 48 h and 72 h after electroporation and stored at -80°C until downstream analysis. Detection was performed by electrochemiluminescence (Meso Scale Discovery Inc.) using a U-PLEX detection kit (IL-15) or V-PLEX detection kits (IFN-γ and TNF-α) according to the manufacturer’s protocol.

### In vitro CD70-CAR NK cell-mediated cytotoxicity of CD70^+^ tumor and CAF target cell lines

Cytotoxic activity toward CD70^+^ tumor cell lines (Raji, PANC-1, and LIM2099) was assessed by co-culturing CD70-CAR, CD70-CAR-IL-15 and MOCK NK cells 24 h after electroporation with PKH67 (Sigma-Aldrich)-labeled CD70^+^ tumor cell lines (Raji, LIM2099, PANC-1) in a 5:1 effector:target ratio in sterile FACS tubes (VWR). After 4 h, co-cultures were stained with 7-AAD and Annexin V-PE (BD Biosciences). Target cell survival was measured on a CytoFLEX flow cytometer and calculated as previously described [34]. To investigate if the observed killing was CAR-mediated, CD70-CAR, CD70-CAR-IL-15, and MOCK NK cells were incubated overnight and during the co-culture with PKH67^+^ Raji cells with 10 μg/mL or 100 μg/mL anti-CD27 neutralizing mAb (Clone MAB382, R&D Systems) or isotype control IgG1κ mAb (clone MAB002, R&D Systems). Co-culture and target cell survival analysis was performed as described above. To evaluate the efficacy of CD70-CAR NK cell-mediated killing after cytokine stimulation, CD70-CAR NK cells were incubated overnight prior to the co-culture with the effector dose 50 (ED50) of IL-2 (0.25 ng/mL), IL-7 (0.50 ng/mL), IL-12 (0.05 ng/mL), IL-15 (2.60 ng/mL) and IL-18 (9.00 ng/mL) cytokines, purchased from R&D Systems. Subsequently, stimulated NK cells were co-cultured with PKH67^+^ Raji, LIM2099 and PANC-1 target cells and analyzed as described above. To exclude if the improved cytotoxic capacity was only due to IL-15 stimulation, CD70-CAR NK cells and MOCK control cells were incubated overnight with IL-15 (ED50: 2.60 ng/mL) and co-cultured with PKH67^+^ LIM2099 cells. Co-culture and target cell survival analysis was performed as described above. Lastly, to investigate the mode of action of IL-15 stimulation, CD70-CAR NK cells were stimulated overnight with the ED50 (2.60 ng/mL) and 20 pg/mL IL-15 (i.e., rounded number of the highest amount of IL-15 measured in the supernatant of CD70-CAR-IL-15 NK cells via electrochemiluminescence), and CD70-CAR expression and cytotoxic capacity were measured 24 h post-electroporation in the same manner as described above.

Longitudinal cytotoxic activity of CD70-CAR, CD70-CAR-IL-15, and MOCK NK cells toward CD70^+^ CAF cell lines (CT5.3hTERT, RLT-PSC, and hPSC21) was analyzed using the xCELLigence Real-Time Cell Analysis (RTCA; Agilent) that records cell viability and growth by impedance measurements. Performance of the NK cell-mediated killing assay with the xCELLigence RTCA was executed as explained previously [35]. In short, seeding density was optimized for each CAF cell line to ensure continuous growth until the end of the assay. CAF cells were seeded in gold-coated 16-well plates and background impedance of the plates was measured before seeding of the target cells. After a 24 h incubation to allow proper adhesion to the plates, CAF cell lines were treated with CD70-CAR, CD70-CAR-IL-15, or MOCK NK cells in a 1:1 effector:target ratio (based on the amount of CAFs seeded at day 0), or left untreated. The impedance signal was monitored by automated measurements every 15 min starting from cell seeding and ending 48 h after treatment. Measurement of the impedance was expressed as Cell Index (CI) and normalized to 1 after starting the co-culture.

### CD70^+^ Raji xenograft mouse model

In vivo efficacy of CD70-CAR, CD70-CAR-IL-15, and MOCK NK cells was evaluated in a subcutaneous Raji xenograft mouse model. Female NSG mice of six weeks old, were obtained from Janvier Labs and maintained at the Animal Core Facilities of the UAntwerp under specific pathogen free conditions in individually ventilated cages enriched with nesting material. All animal procedures were conducted in accordance with, and approval of, the Animal Ethics Committee of the UAntwerp under registration number 2021–55. Mice were injected subcutaneously with 1.0 × 10^6^ Raji cells suspended in 100 μL sterile PBS (Life Technologies) containing 12 mg/mL Geltrex (Life Technologies) at the left abdominal flank. Tumor growth was measured using a digital caliper and tumor area was assessed by measuring length and width, and calculated using the formula ‘tumor area = length x width’. When tumors reached 50 mm^2^, mice were randomized based on tumor size into four different treatment groups (day 0): (1) untreated, (2) MOCK NK cells, (3) CD70-CAR NK cells and (4) CD70-CAR-IL-15 NK cells. All mice, except for the untreated group, were treated on day 0 and day 4 intravenously (i.v.) via the tail vein with 1.0 × 10^7^ CD70-CAR, CD70-CAR-IL-15, or MOCK NK cells suspended in 200 μL sterile PBS. Prior to injection, CD70-CAR, CD70-CAR-IL-15, and MOCK NK cells were irradiated 4 h post-electroporation with a sublethal radiation dose of 10 Gy using the X-RAD 320 irradiation device (Precision X-ray Inc.) at a concentration of 4.0 × 10^6^ cells/mL in sterile culture flasks. Dosimetry was performed using Gafchromic EBT3 films, irradiations were performed from the top of the culture flasks and the dose was prescribed at the exit. Mice were sacrificed when tumor size reached 150 mm^2^.

### CD70-CAR NK cell-mediated cytotoxicity of CD70^+^ microtumors

Longitudinal cytotoxic activity of (CAR) NK cells toward CD70^+^ CAFs in an advanced in vitro model was evaluated using 3D patient-derived PDAC microtumors containing PDAC tumor organoids with RLT-PSC CAF cells. Patient-derived PDAC organoids (P002, P044 and P87) from our in-house organoid bank, were cultured as previously described [36, 37]. In short, for passaging organoids were digested into single cell suspension using TrypLE (Life Technologies) and plated in 100% cultrex (Bio-Techne Ltd) drops on flat bottom culture plates (Greiner bio-one). The drops were covered with Advanced DMEM/F12 medium (Life Technologies) supplemented with 1% GlutaMAX (Life Technologies), 1% HEPES (Life Technologies), 1% P/S, 4% Noggin-Fc Fusion Protein (ImmunoPrecise Antibodies), 4% R-Spondin-Fc Fusion Protein (ImmunoPrecise Antibodies), 1 × B27 supplement (Life Technologies), 1.25 N-Acetylcysteine (Sigma-Aldrich), 10 mM Nicotinamine (Sigma-Aldrich), 500 nM A83 (Bio-Techne), and 5 nM Wnt Surrogate-Fc Fusion Protein (ImmunoPrecise Antibodies).

For downstream microtumor generation, organoids were mixed with red fluorescent-transduced RLT-PSC cells, seeded in Advanced DMEM/F12 medium containing 3% Cultrex, supplemented with 1% GlutaMAX, 1% HEPES, and 1% P/S in 384-well ultra-low attachment microplates (Corning), and incubated at 37°C for two days to allow assembling of microtumors. On day three, microtumors were treated with CD70-CAR, CD70-CAR-IL-15, or MOCK NK cells in a 1:1 effector:target ratio (based on the amount of RLT-PSC cells seeded on day 0) or left untreated and followed up by the Tecan Spark Cyto live-cell imager every 2 h for 36 h. Brightfield and fluorescent images were analyzed using our in-house developed analysis software Orbits [38] and growth rate of RLT-PSC cells in the microtumors was determined by normalizing the fluorescent red signal (sum red area) to the first measurement (T0) or the control (untreated) at the respective timepoint (12 h, 24 h, 36 h).

### Data analysis

FlowJo v10.8.2 Software (BD Life Sciences) and ImageJ v1.53t (U. S. National Institutes of Health) were used for flow cytometry data and microtumor image analysis, respectively. Prism version 9.1.2 (GraphPad) was used for graphical data representation. Statistical computations were performed in JMP Pro v16.0.0 (SAS Institute Inc.). Linear Mixed Models were used to compare means between two or more groups. In the case of comparing the means of more than two groups and the null hypothesis was rejected by the Fixed Effects test, a multiple comparison post hoc analysis was applied. Dunnett’s correction for multiple comparison was used when comparing to a control sample. For all pairwise comparisons, Tukey’s multiple comparison correction was applied. A nonlinear Spearman’s correlation with a simple linear regression was done to analyze the correlation between CD70-CAR and CD70-CAR-IL-15 NK-mediated cytotoxicity and CD70 target expression. Differences in probability of survival between treatments groups in in vivo experiments were analyzed using the Log-Rank (Mantel-Cox) test with the SPSS Statistics v28.0.1.1. software (IBM). To analyze differences in tumor kinetics over time, we used R with the “afex” and “emmeans” [39] packages to perform mixed model ANOVAs. Differences were considered to be significantly different if *p*-value < 0.05 (*p < 0.05; **p < 0.01; ***p < 0.001; and ****p < 0.0001). Error bars represent mean values ± standard deviation (SD) unless stated otherwise.

## Results

### CD70 is an attractive therapeutic target on both tumor cells and CAFs in CRC and PDAC patients

**Fig. 1:**
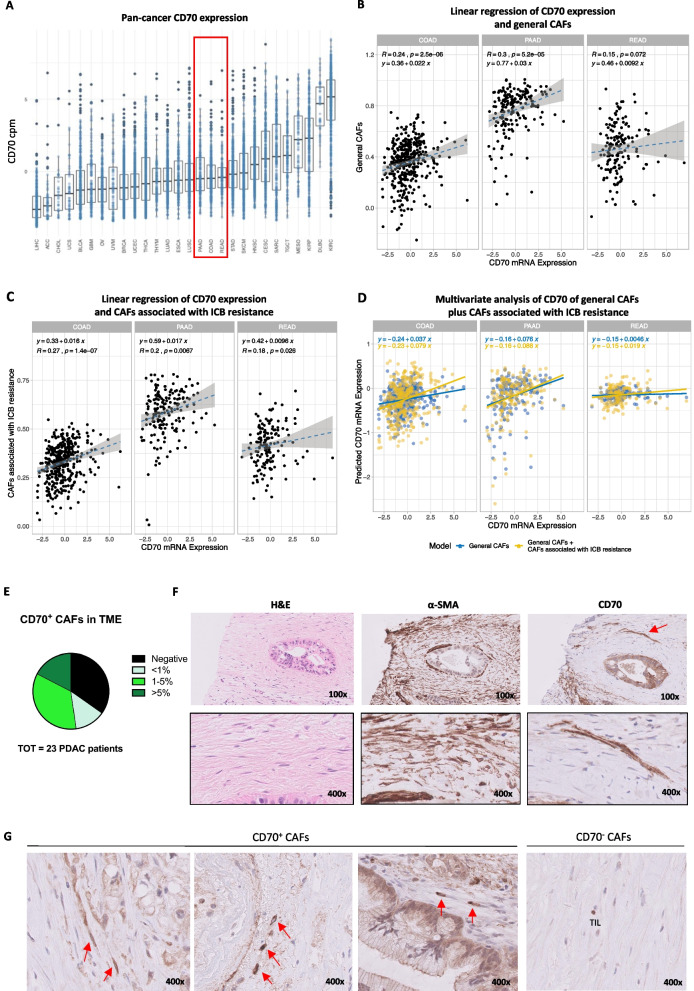
CD70 expression in CRC and PDAC patients.** A** Pan-cancer CD70 expression in count per million reads (cpm) obtained from bulk RNA-seq of 28 different cancer types from the PCAWG Firehose cohort from the TCGA database. **B, C** Linear regression analysis and Spearman’s correlation analysis of CD70 mRNA expression and the general CAF score on CRC (i.e., COAD/READ) and PDAC (i.e., PAAD) databases, respectively. **D** Multivariate regression analysis with log ratio test to predict CD70 using the general CAF score (blue) and CAF score related to ICB therapy resistance on top of the general CAF score (yellow). **E** Distribution of percentage CD70^+^ CAFs of the total stromal fibroblasts found in the TME of 23 PDAC patients. **F** Representative images of H&E, α-SMA, and CD70 staining of a PDAC patient. **G** Representative images of CD70^+^ CAFs (indicated by red arrows) in the stromal compartment and an area with CD70^−^ CAFs in the stromal compartment with a CD70^+^ TIL as reference. Total magnification is indicated in the lower right corner. Abbreviations: CRC, colorectal cancer; PDAC, pancreatic ductal adenocarcinoma; CAF, cancer-associated fibroblast; ICB, immune checkpoint blockade; TME, tumor microenvironment; H&E, hematoxylin and eosin; α-SMA, alpha-smooth muscle actin; and TIL, tumor-infiltrating lymphocyte

CD70 has been postulated as a putative pan-cancer target for immunotherapy, which is supported by bulk RNA-seq data from the TCGA PCAWG Firehose cohort demonstrating varying CD70 mRNA expression levels across 28 different cancer types (Fig. 1A). Focusing on CRC (i.e., COAD/READ) and PDAC (i.e., PAAD) patients, intermediate CD70 mRNA expression levels were observed (Fig. 1A). Our research and others in CRC indicated that CD70 expression is more abundantly found on CAFs residing in the TME, rather than on tumor cells [19, 20]. Since PDAC is characterized by large amounts of CAFs, we postulated that CD70-expressing CAFs could be present in PDAC patients as well. To analyze the relationship between CD70 mRNA expression and presence of CAFs, we used published gene sets identifying general CAFs and CAFs associated with immune checkpoint blockade (ICB) therapy resistance to estimate their numbers in CRC (i.e., COAD/READ) and PDAC (i.e., PAAD) [26]. Confirming our hypothesis, linear regression analysis indicated that the presence of both groups of CAFs is associated with the CD70 mRNA expression, especially in COAD and PAAD (Fig. 1B, C). In addition, association with CD70 mRNA expression is stronger for CAFs associated with ICB therapy resistance in COAD (*R*_CAF_ = 0.24 vs. *R*_ICB CAF_ = 0.27) and READ (*R*_CAF_ = 0.15 vs. *R*_ICB CAF_ = 0.18; Fig. 1C). These results were also recapitulated when performing Spearman’s correlation analysis (Fig. 1B, C). A multivariate model containing estimates of both general CAFs and CAFs associated with ICB therapy resistance performed even better in predicting CD70 mRNA expression as compared to a univariate model of general CAFs alone in all three data sets (Fig. 1D), but significance was only reached in COAD (*P*_COAD_ < 0.001; *P*_READ_ = 0.145; *P*_PAAD_ = 0.128).

CD70 expression on CAFs in the TME of PDAC was confirmed at protein level in a cohort of 23 PDAC patients (Fig. 1E, F, G) and an extra cohort of 9 PDAC patients (Additional file 1: Figure S1A). CD70^+^ CAFs (i.e., α-SMA^+^ regions) were observed in 65% (15/23) of the total cohort of which CD70^+^ CAFs were abundantly present (i.e., 1–5%) in 34.8% (8/23) and very high (i.e., > 5%) in 17.4% (4/23) of the PDAC patients (Fig. 1C and Additional file 1: Figure S1A). Although these findings are based on a limited sample size, it is noteworthy that a high amount of CD70^+^ CAFs (i.e., 1–5% and > 5%) appears to be more prevalent in advanced disease stages (Additional file 1: Figure S1B). In line with what is reported in literature, no other cell types, except for a few activated tumor-infiltrating lymphocytes, were found positive for CD70 in the TME. Additionally, PBMCs from healthy donors showed low to absent CD70 expression on circulating CD4^+^ and CD8^+^ T cells, B cells, NK cells and monocytes (Additional file 1: Figure S1C), highlighting the restricted expression pattern of CD70 on healthy tissue.

Our results indicate that CD70 is an attractive therapeutic target expressed on both cancer cells and CAFs in CRC and PDAC patients.

### CD70-CAR NK cells show competent antigen-specific lysis of CD70^high^-expressing tumor cells

**Fig. 2:**
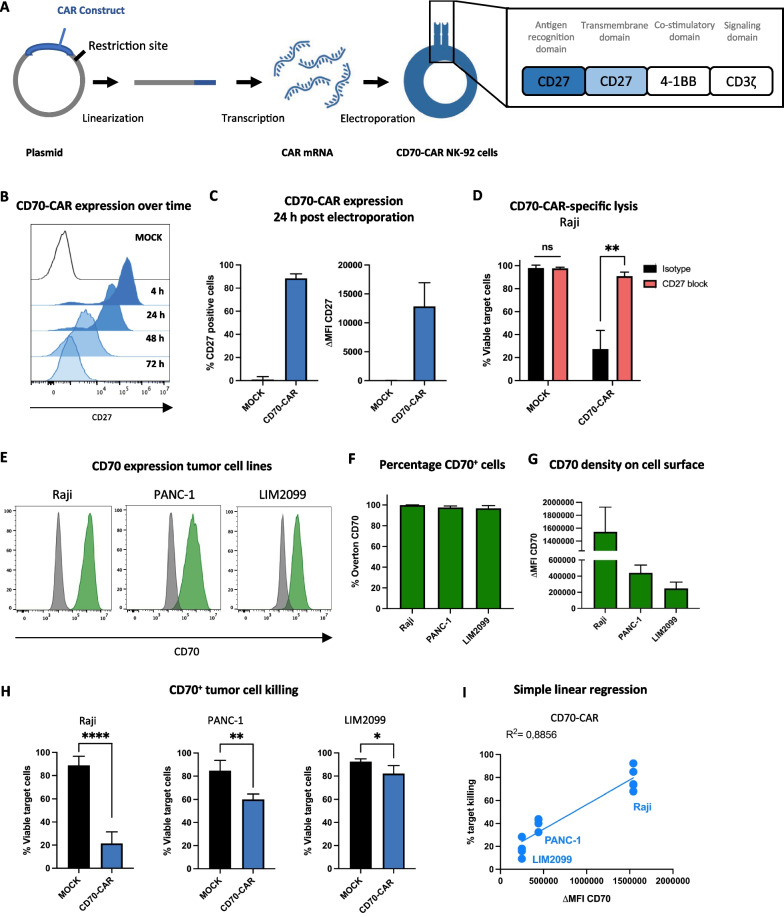
CD70-CAR NK cell development and validation. **A** Schematic representation of CD70-CAR NK cell generation and structural composition of the CD70-CAR construct. **B** CD70-CAR expression was detected by measuring CD27 expression on the cell surface using flow cytometry. Representative histograms of CD27 expression on NK-92 cells 4 h, 24 h, 48 h and 72 h after electroporation without (MOCK; white) or with CAR-encoding mRNA (CD70-CAR; blue). **C** Quantification of the amount CD27^+^ cells and the intensity of CD27 expression, depicted as mean fluorescence intensity minus isotype control (ΔMFI), 24 h post-electroporation (*n =* 6). **D** Percentage of viable Raji cells after a 4 h co-culture with CD70-CAR NK cells or MOCK control cells in the presence of 10 μg/mL anti-CD27 blocking antibody (CD27-block; red) or corresponding isotype control (Isotype; black; *n =* 4). **E** Representative flow cytometry histograms of CD70 expression on tumor cell lines (Raji, PANC-1, and LIM2099). **F**-**G** Quantification of percentage CD70^+^ cells and intensity of CD70 expression (ΔMFI) for Raji, PANC-1, and LIM2099 tumor cell lines, respectively. **H** Percentage of viable CD70^+^ tumor cells (Raji, PANC-1 and LIM2099) after a 4 h co-culture with CD70-CAR NK cells or MOCK control cells (*n =* 5). **I** Simple linear regression analysis of CD70-CAR NK cell target lysis and density of CD70 expression (ΔMFI) on target cells. Spearman’s correlation was used to analyze the correlation between the CD70 expression and target lysis. Linear mixed models were used to compare means of the lysis of the different tumor targets. ns = *p* > 0.05; *p < 0.05, **p < 0.01 and ****p < 0.0001

We hypothesized that CD70-expressing tumor cells and CAFs could be effectively eliminated by CAR NK cells. Therefore, a second-generation CD70-targeting CAR construct was developed consisting of the extracellular and transmembrane domains of the natural CD27 receptor, a 4-1BB-derived co-stimulatory domain, and a CD3ς-derived signaling domain. NK-92 cells were used as NK cell source and were electroporated with CD70-CAR mRNA to obtain CD70-CAR NK cells (Fig. 2A).

Expression of the CD70-CAR construct on the cell surface was analyzed with flow cytometry by staining for CD27 (Additional file 1: Figure S2A). Considering the possible basal expression of CD27 in NK cells, NK-92 cells electroporated without CAR mRNA (i.e., MOCK) were used as control cells to determine CAR-specific CD27 expression. Only negligible basal CD27 expression (Overton_24h_: 1.0 ± 2.4%) was detected on MOCK NK cells (Fig. 2C and Additional file 1: Figure S2A, B). On the other hand, CD70-CAR NK cells displayed maximal CD27 expression already 4 h after mRNA electroporation, maintained high CD27 expression 24 h post-mRNA electroporation (Overton_24h_: 88.5 ± 3.8%, ΔMFI_24h_: 12,848.3 ± 4,097.8) and reached baseline CD27 expression levels 72 h post-electroporation (Fig. 2B, C). To assess target-specific cytotoxicity, CD70-CAR NK cells were co-cultured with the NK cell-resistant Raji cell line in the presence or absence of a CD27-neutralizing mAb. CD70-CAR NK cells were indeed capable of eradicating Raji cells predominantly in a CAR-mediated manner (Viable Raji_isotype_: 27.5 ± 16.2% vs Raji_CD27-block_: 90.9 ± 3.5%; Fig. 2D, Additional file 1: Figure S2C). Cytotoxic capacity of CD70-CAR NK cells was further evaluated against three > 95% CD70^+^ hematological and solid tumor cell lines, of which a Burkitt’s lymphoma-derived cell line (Raji; ΔMFI_CD70_: 15,429,891.3 ± 383,907.9) displayed highest CD70 surface density, a PDAC-derived cell line (PANC-1; ΔMFI_CD70_: 440,510.0 ± 96,692.9) intermediate CD70 density and a CRC cell line derived from a metastatic lesion (LIM2099; ΔMFI_CD70_: 248,192.0 ± 77,270.6) low CD70 density (Fig. 2E, F, G). CD70-CAR NK cells eliminated all three cell lines significantly better than MOCK control cells (Fig. 2H). Interestingly, maximal killing was detected in Raji cells (Viable Raji: 21.5 ± 9.9%), where CD70 expression was high, and killing was limited in the lowest CD70 expressing cell line (Viable LIM2099: 82.1 ± 6.9%; Fig. 2I). A positive linear relation (*R*^2^ = 0.8856) was observed between CD70-CAR NK cell-mediated killing and the CD70 antigen density on target cells, although not statistically significant different presumably due to a low sample size (*p* = 0.333).

These findings illustrate that the generated CD70-CAR NK cells are competent in antigen-specific CD70^+^ tumor cell lysis, yet our data strongly suggest that effective elimination is only achieved when the CD70 antigen is abundantly expressed.

### Interleukin 15 enhances the functionality of CD70-CAR NK cells

**Fig. 3:**
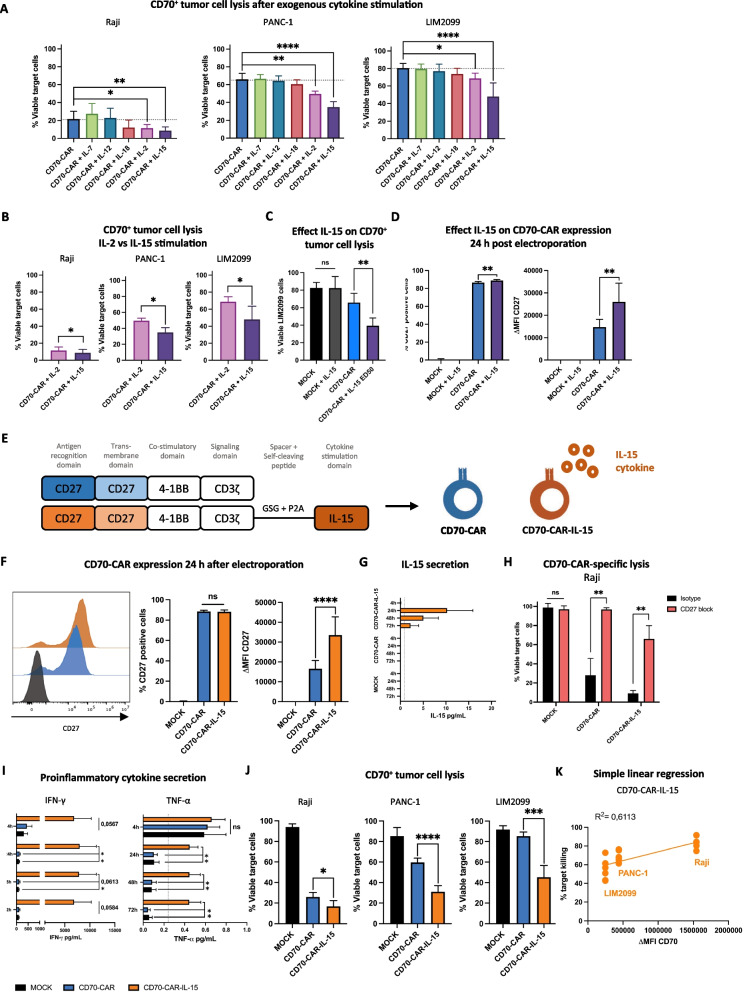
Effect of IL-15 cytokine stimulation on CD70-CAR NK cell functionality. **A** Percentage viable CD70^+^ target cells after a 4 h co-culture with unstimulated CD70-CAR NK cells or stimulated overnight with IL-2, IL-7, IL-12, IL-15, or IL-18 cytokines (*n =* 4). **B** Percentage viable CD70^+^ target cells after a 4 h co-culture with CD70-CAR NK cells stimulated overnight with IL-2 or IL-15 (*n =* 4). **C** Percentage viable LIM2099 cells after a 4 h co-culture with unstimulated MOCK or CD70-CAR NK cells or stimulated overnight with IL-15 (*n =* 5).** D** Quantification of CD27^+^ cells and intensity of CD27 expression (ΔMFI), 24 h after electroporation on CD70-CAR NK cells or MOCK cells cultured overnight with or without IL-15 (*n =* 5). **E** Structural design of CD70-CAR and CD70-CAR-IL-15 constructs. **F** Representative histograms and quantification of surface CD27 expression on MOCK NK cells (black), CD70-CAR NK cells (blue) and CD70-CAR-IL-15 NK cells (orange) 24 h after electroporation (*n =* 8). **G** Secreted IL-15 in the supernatant of MOCK, CD70-CAR NK cells, or CD70-CAR-IL-15 NK cells after electroporation (*n =* 3). **H** Percentage viable Raji cells after a 4 h co-culture with CD70-CAR NK cells, CD70-CAR-IL-15 NK cells or MOCK cells in the presence of 100 μg/mL anti-CD27 blocking antibody or isotype control (*n =* 4). **I** Secreted IFN-γ, and TNF-α in the supernatant of MOCK, CD70-CAR NK cells, or CD70-CAR-IL-15 NK cells after electroporation (*n =* 6). Error bars represent mean ± standard error of mean. **J** Percentage viable target cells after a 4 h co-culture with CD70-CAR NK cells, CD70-CAR-IL-15 NK cells or MOCK cells (*n =* 6). **K** Simple linear regression and Spearman Correlation analysis of CD70 ΔMFI and CD70-CAR-IL-15 target cell lysis. Linear mixed models with either Dunnett’s or Tukey’s correction for multiple comparison was applied to compare means. ns = *p* > 0.05; *p < 0.05, **p < 0.01, ***p < 0.001 and ****p < 0.0001

Since cytokines play an important role in regulating the effector functions of NK cells, we explored the impact of cytokine stimulation on ameliorating the cytotoxic activity of CD70-CAR NK cells. CD70-CAR NK cells were cultured overnight with cytokines that were previously described to improve CAR T or NK cell product potency, being IL-2, IL-7, IL-12, IL-15, and IL-18, and co-cultured them with CD70^+^ tumor cell lines (Raji, PANC-1, and LIM2099 cells). Stimulation with IL-2 or IL-15 resulted in significantly increased cytotoxic activity toward all three tumor cell lines (Fig. 3A), of which IL-15 demonstrated significantly greater target cell lysis than IL-2 in all three target cell lines (Viable Raji: 11.4 ± 4.1% vs 8.7 ± 4.1%, viable PANC-1: 49.6 ± 3.1% vs 34.8 ± 6.0%, and viable LIM2099: 68.7 ± 5.8% vs 47.9 ± 15.6%, respectively; Fig. 3B). Furthermore, IL-15 stimulation alone did not improve NK cell-mediated killing but required the concurrent presence of the CD70-CAR (Viable LIM2099_MOCK + IL-15_: 82.4 ± 13.3% vs LIM2099_CD70-CAR_: 65.9 ± 10.6% vs LIM2099_CD70-CAR + IL-15_: 39.4 ± 8.9%; Fig. 3C). Exogenous stimulation with IL-15 also resulted in a slightly higher frequency of CD70-CAR^+^ NK cells and an almost double CAR surface density (ΔMFI: 14,722.6 ± 3,432.8 vs. 26,018.6 ± 8,317.3, respectively; Fig. 3D).

Building on these encouraging results, we developed a fourth-generation CD70-targeting CAR that incorporates an IL-15 cytokine cassette in the CD70-CAR mRNA construct, separated by a GSG spacer and a P2A self-cleaving peptide to obtain equimolar co-expression of the CD70-CAR and the IL-15 cytokine (CD70-CAR-IL-15; Fig. 3E). Validation of CAR expression from CD70-CAR-IL-15 construct showed improved CAR density compared to the CD70-CAR construct 24 h post-electroporation (ΔMFI_24h_: 16,582.1 ± 4,127.4 vs 33,538.5 ± 9,162.4, respectively; Fig. 3F), in line to the exogenous addition of IL-15 (Fig. 3D). IL-15 stimulation even extended the amount of CD70-CAR-expressing NK cells and intensity of the expression over time, observed at 48-h post-electroporation and to some extent 72-h post-electroporation (Additional file 1: Figure S3A). Additionally, low concentrations of IL-15 were detected in the supernatant of CD70-CAR-IL-15 NK cell cultures which peaked at 24-h post-electroporation (10.3 ± 5.7 pg/mL; Fig. 3G). Functionally, CD70-CAR-IL-15 NK cells demonstrated primarily CAR-mediated killing of CD70^+^ target cells (Viable Raji_isotype_: 9.1 ± 3.0% vs Raji_CD27-block_: 66.0 ± 12.1%; Fig. 3H). Although we only detected low levels of IL-15, the activation status of CD70-CAR NK cells was drastically augmented through elevated secretion of proinflammatory cytokines IFN-γ (at 24 h: 133.1 ± 33.7 pg/mL vs. 7,954.6 ± 3,321.6 pg/mL, respectively) and TNF-α (at 24 h: 0.10 ± 0.03 pg/mL vs 0.45 ± 0.08 pg/mL, respectively; Fig. 3I), and through upregulation of activating receptor DNAM-1 and downregulation of inhibitory receptor TIGIT compared to MOCK and CD70-CAR NK cells (Additional file 1: Figure S3B). Interestingly, expression of PD-1 and LAG3, a negative regulator of NK cytokine production, remained unchanged upon IL-15 stimulation (Additional file 1: Figure S3B). This improved activation was also converted to improved killing of CD70^+^ tumor cell lines compared to CD70-CAR NK cells (Viable Raji: 16.8 ± 5.5% vs 25.9 ± 4.5%, viable PANC-1: 31.1 ± 5.9% vs 59.6 ± 4.2%, viable LIM2099: 45.3 ± 11.5% vs 85.5 ± 3.9%, respectively; Fig. 3J). In fact, a simple linear regression indicated that the killing activity of CD70-CAR-IL-15 NK cells depended less on CD70 target density (*R*^2^ = 0.6113; Fig. 3K) compared to conventional CD70-CAR NK cells (*R*^2^ = 0.8856, Fig. 2I). Furthermore, we unraveled that IL-15 produced by CD70-CAR-IL-15 NK cells had a (partial) autocrine or intracellular mode of action (Additional file 1: Figure S3C). This was inferred after conventional CD70-CAR NK cells were stimulated overnight with either a high concentration of IL-15 (ED50: 2.60 ng/mL) or the rounded amount of the highest concentration of IL-15 detected in cultures of CD70-CAR-IL-15 NK cells (i.e., 20 pg/mL; Fig. 3G). While CAR expression nor target killing capacity was significantly different from CD70-CAR-IL-15 cells when CD70-CAR NK cells were stimulated with a high amount of exogenous IL-15, CD70-CAR NK cells supplemented with the same amount of IL-15 as secreted by CD70-CAR-IL-15 NK cells showed significant lower CAR density (ΔMFI_CD70-CAR-IL-15_: 29,816.0 ± 14,060.6 vs ΔMFI_CD70-CAR + IL-15 SN_: 14,763.4 ± 4,362.3) and target cell killing (Viable LIM2099_CD70-CAR-IL-15_: 32.2 ± 14.6% vs LIM2099_CD70-CAR + IL-15 SN_: 61.6 ± 10.9%; Additional file 1: Figure S3C).

Altogether, CD70-CAR NK cells armored with IL-15 have superior cytotoxic activity over conventional CD70-CAR NK cells, which is fueled by an increased CAR density and a boosted activation status.

## IL-15 is required to maintain CD70-CAR efficacy in vivo

**Fig. 4:**
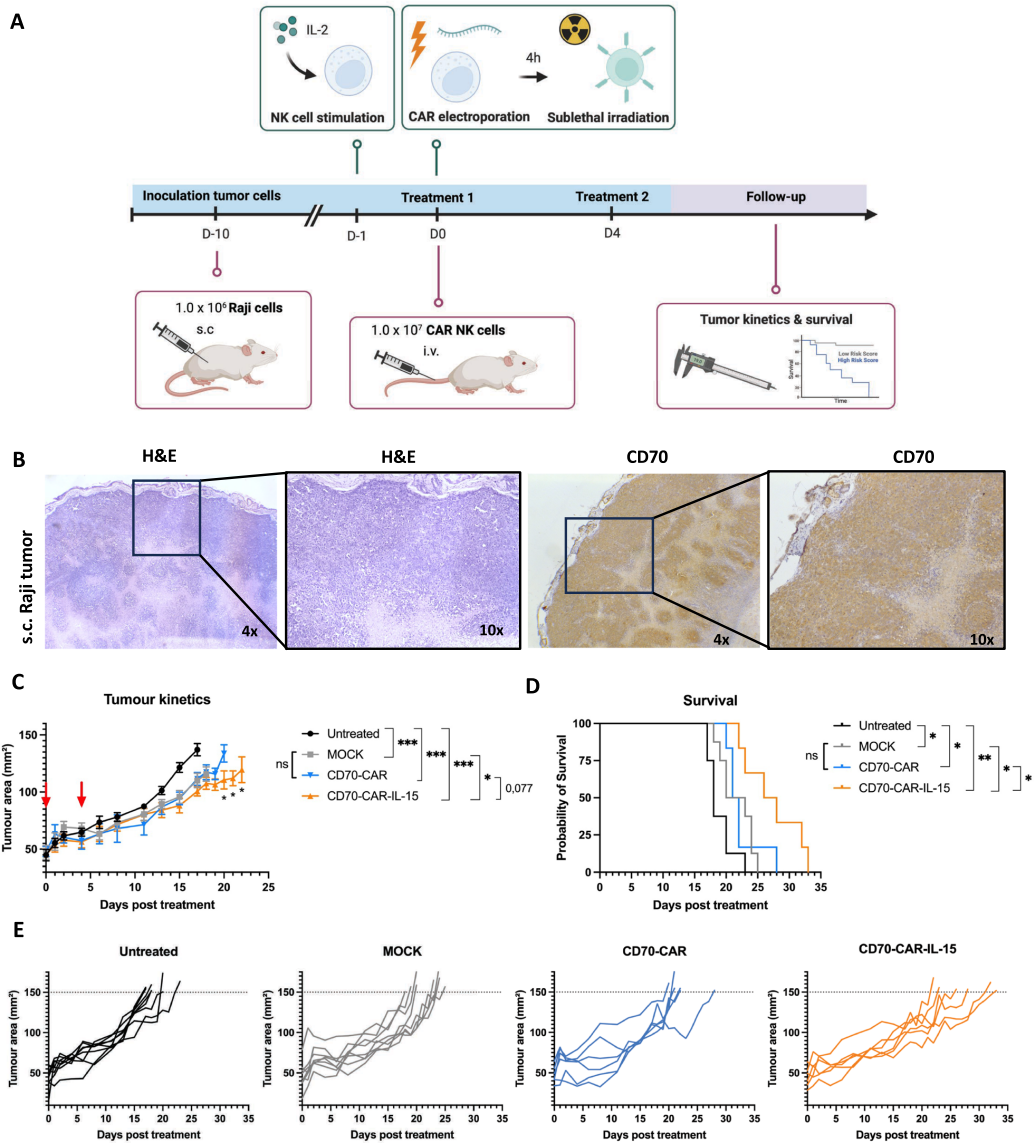
Tumor kinetics and survival of subcutaneous CD70^+^ Raji-bearing mice after treatment with CD70-CAR NK cells and CD70-CAR-IL-15 NK cells. **A** Schematic representation of (top) production of MOCK NK cells, CD70-CAR NK cells, and CD70-CAR-IL-15 NK cells prior to injection, and (bottom) schedule of generation and treatment of the CD70^+^ Raji xenograft mouse model. **B** Representative images of FFPE-slides of a Raji tumor stained for H&E and for CD70 positivity. Raji-bearing mice were treated twice with three days in between with 1.0 × 10^7^ MOCK NK cells (*n =* 8), CD70-CAR NK cells (*n =* 6) or CD70-CAR-IL-15 NK cells (*n =* 6). Untreated mice were included as control (*n =* 8). **C** Tumor kinetics over time post-treatment; red arrows indicate treatment days. Error bars represent mean values ± standard error of mean. **D** Survival curve post-treatment. **E** Separated spider plots of the tumor kinetics over time per treatment group. Mixed model ANOVA was used to compare differences in tumor kinetics and the Log-Rank (Mantel-Cox) test was performed to analyze differences in survival. *p < 0.05, **p < 0.01 and ***p < 0.001

In order to validate whether CD70-CAR-IL-15 NK cells maintained an improved functionality in a systemic setting, we established a xenograft, subcutaneous CD70^+^ Raji-bearing mouse model using NSG mice (Fig. 4A, B). NSG mice were subcutaneously injected with 1.0 × 10^6^ Raji cells mixed with Geltrex and when tumor size reached 50 mm^2^, mice were treated twice (three days in between) with 1.0 × 10^7^ MOCK control NK cells, CD70-CAR NK cells, or CD70-CAR-IL-15 NK cells or left untreated (Fig. 4A). To prevent engraftment of NK-92 cells, (CAR) NK cell therapies were irradiated with a 10 Gy sublethal dose right before i.v. injection, which did not affect CAR expression (Additional file 1: Figure S4A). After injection, a transient drop in body weight was observed in the groups treated with (CAR) NK cells that rapidly recovered to baseline (Additional file 1: Figure S4B). All three NK cell therapies (MOCK, CD70-CAR, and CD70-CAR-IL-15) significantly delayed tumor growth over time and improved survival compared to the untreated control group (Median survival untreated: 18 days, MOCK: 21.5 days, CD70-CAR: 21.5 days, and CD70-CAR-IL-15: 27 days; Fig. 4C, D, E). Yet, treatment with CD70-CAR NK cells did not result in a significant difference in tumor growth or survival compared to treatment with MOCK NK cells (Figure C, D). Hence, the improved cytotoxic activity of CD70-CAR NK cells over MOCK NK cells observed against Raji cells in vitro (Fig. 2D, E) was not translated to an in vivo setting. On the other hand, CD70-CAR-IL-15 NK cells induced a significant decrease in tumor burden compared to MOCK NK cells and a trend compared to conventional CD70-CAR NK cells over time (*significant on days 20, 21 and 22 post-treatment; Fig. 4C). Consistent with tumor kinetics, CD70-CAR-IL-15 NK cells significantly improved survival of Raji-bearing mice (Fig. 4D).

Taken together, these results imply that stimulation with IL-15 is a prerequisite for CD70-CAR NK cells to obtain effective potency against CD70^+^ tumors in vivo.

## CD70^+^ CAFs are effectively eliminated over time by IL-15 armored CD70-CAR NK cells

**Fig. 5:**
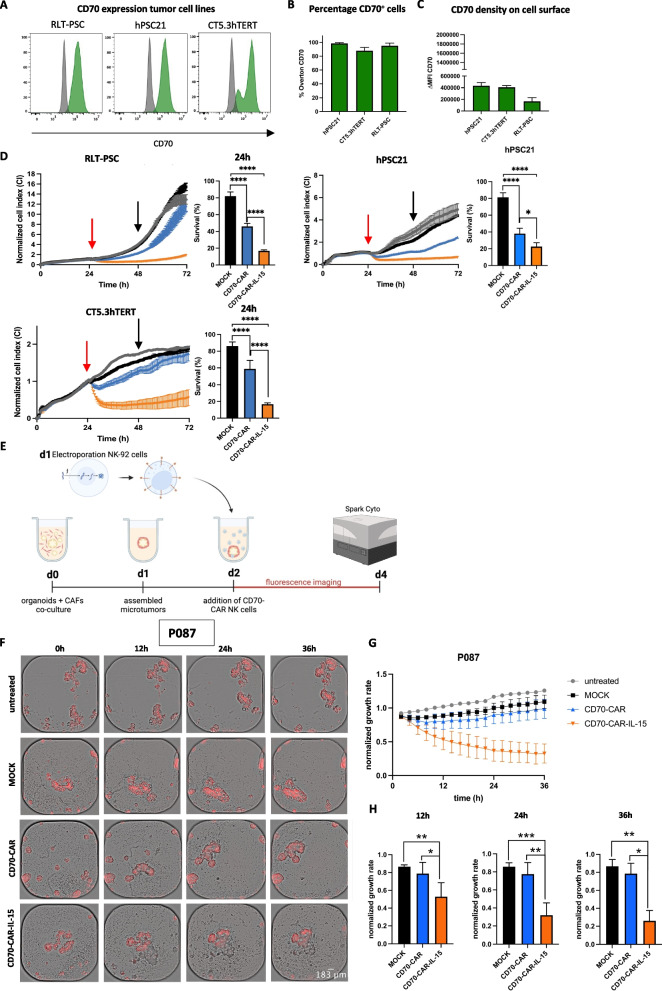
Cytotoxic activity of CD70-CAR NK cells and CD70-CAR-IL-15 NK cells against CD70^+^ CAFs. **A-C** CD70 expression on RLT-PSC, hPSC21, and CT5.3hTERT CAF cell lines. **A** Representative flow cytometry histograms. **B-C** Quantification of percentage CD70^+^ cells and intensity of CD70 expression (ΔMFI). **D** Representative graphs of longitudinal survival follow-up (i.e., Cell Index; CI) with the xCELLigence RTCA system and corresponding quantification after 24 h co-culture (indicated with black arrow; normalized to untreated) of RLT-PSC (*n =* 6), hPSC21 (*n =* 3), and CT5.3hTERT (*n =* 4) CAF cell lines with CD70-CAR NK cells, CD70-CAR-IL-15 NK cells, or MOCK NK cells. Monocultures of CAF cell lines treated with culture medium were used as control. To ensure proper adhesion, CAF cell lines were grown over 24 h after which treatment with (CAR) NK cells started (indicated by a red arrow) and the CI was followed up 48-h post-treatment. **E** Schematic representation of the experimental setup of PDAC patient-derived microtumors, containing tumor organoids with fluorescent red labelled RLT-PSC cells, in co-culture with (CAR) NK cells for two days. Monocultures of microtumors treated with medium were included as control. Follow up using live cell imaging was done using the Spark Cyto multimode reader. **F–H** Co-cultures of PDAC patient 087 microtumors (P87) with different treatment conditions: untreated, MOCK control NK cells, CD70-CAR NK cells, and CD70-CAR-IL-15 NK cells. **F** Representative brightfield images overlayed with the red fluorescent signal. **G** Growth rate, normalized against T0h, over time for the different treatment conditions. **H** Quantification of three different timepoints (12-h, 24-h, and 36-h post-treatment), comparing the treatment conditions normalized to the untreated control at that timepoint (*n =* 3). Images were cropped with ImageJ. Error bars represent mean values ± standard error of mean. Linear mixed models with Tukey’s correction for multiple comparison were applied to compare means of cell survival. * *p* < 0.05, **p < 0.01, ***p < 0.001 and ****p < 0.0001

Given the pro-tumorigenic nature of CD70^+^ CAFs, we examined whether these could be targeted by CD70-directed CAR NK cells. Therefore, CD70^+^ immortalized CAF cell lines, derived from PDAC patients (i.e., RLT-PSC and hPSC21) and a CRC patient (i.e., CT5.3hTERT; Fig. 5A, B, C) were co-cultured with CD70-CAR NK cells, CD70-CAR-IL-15 NK cells, or MOCK control NK cells, and longitudinal CAF cell survival was monitored using the xCELLigence RTCA system. In line with our previous in vitro data on tumor cells, CD70-CAR NK cells showed improved reduction of CAF growth over time compared to MOCK NK cells in all three CAF cell lines. Yet, CD70-CAR-IL-15 NK cells were significantly more competent than CD70-CAR NK cells in completely eradicating CD70^+^ PDAC and CRC CAFs (RLT-PSC_24h_: 13.3 ± 1.5% vs 66.2 ± 5.2%, hPSC21_24h_: 16.9 ± 1.2% vs 45.9 ± 3.6%, CT5.3hTERT_24h_: 16.7 ± 1.6% vs 58.7 ± 10.2%, respectively; Fig. 5D).

These results were validated in more advanced 3D microtumors, established from three different PDAC patient-derived organoids (P002, P044, P087) together with CD70^+^ RLT-PSC CAFs. Patient-derived microtumors harbor intrinsic and inter-patient variability, and recapitulate the stromal fibroblasts. Organoids were cultured with red fluorescent RLT-PSC cells to assemble microtumors, and treated after two days with MOCK control NK cells, CD70-CAR NK cells, CD70-CAR-IL-15 NK cells or left untreated. Subsequently, the red fluorescent signal was monitored over time by live-cell imaging using the Spark Cyto multimode plate reader (Fig. 5E). Analysis of brightfield images overlayed with the red fluorescence signal showed that MOCK NK and CD70-CAR NK cell therapies engage with microtumors of all three patients, but were only able to slightly slow down CAF growth. In contrast, engagement of CD70-CAR-IL-15 NK cells with PDAC microtumors resulted in a reduction of the amount of CAFs over time (Fig. 5G, H; Additional file 1: Figure S5A, B, D, E). Quantification of the CAF normalized growth rate at different time points after treatment demonstrated a slight decrease in CAFs by CD70-CAR NK cells compared to MOCK control cells at most timepoints, although never significant. Of note, CD70-CAR-IL-15 NK cells significantly decreased the amount of CAFs in all three patients at all timepoints compared to MOCK NK cells and CD70-CAR NK cells (normalized growth rate P002_36h_:0.48 ± 0.11 vs 0.76 ± 0.10, P044_36h_: 0.24 ± 0.09 vs 0.80 ± 0.09, P087_36h_: 0.26 ± 0.11 vs 0.79 ± 0.11, respectively; Fig. 5H; Additional file 1: Figure S5C, F). Confirming inter-patient variations, differences in response to CD70-CAR-IL-15 NK cells were observed over the three PDAC patients. While P044 responded best toward CD70-CAR-IL-15 NK cell therapy, P002 showed the least response (mean MOCK normalized growth rate: P044_36h_: 0.30 ± 0.11, P002_36h_: 0.58 ± 0.11; Additional file 1: Figure S5G).

With these data we provide evidence CD70^+^ CAFs can be targeted by CAR-engineered NK cells and that IL-15 stimulated CD70-CAR NK cells demonstrate sustained elimination of these cells over time.

## Discussion

Here, we demonstrated in vitro and in vivo the therapeutic potential of IL-15 armored CD70-CAR NK cells to eliminate both CD70^+^ tumor cells and, most interestingly, also tumor-promoting CD70^+^ CAFs in CRC and PDAC.

Development of CAR-based cell therapies has mainly showed success in hematological tumors, especially with CD19-CAR T cells in B cell malignancies [40–42]. Obtaining positive clinical outcomes in solid tumors like CRC and PDAC has been more challenging due to (1) heterogeneous expression of candidate surface antigens, (2) a more complicated homing to the tumor site, and (3) immune suppression by the hostile TME, including strong stromal barriers consisting of high amounts of CAFs [21, 43]. In this regard, the immune checkpoint molecule CD70 is an interesting candidate with expression in a wide array of solid tumors, nearly absent on healthy tissue, and an apparent involvement in anti-tumor immune suppression [6, 7].

With regard to other tumor types, CRC and PDAC patients displayed a median CD70 expression when evaluating CD70 expression pan-cancer on RNA-seq data available through the TCGA database. This is similar to what has been reported so far [1, 8, 19, 20]. Interestingly, gene set enrichment analysis [26] in CRC and PDAC patients indicated that CD70 expression was also associated with CAFs residing in the tumor stroma. It even suggested that CD70^+^ CAFs in CRC and PDAC are related to ICB resistance, although functional research for this is necessary. Confirmation of CD70 expression on protein level in CAFs has previously been done by our group in CRC and others [19, 20]. In this study, we certified via immunohistochemical analysis the presence of CD70^+^ CAFs in PDAC patient samples as well. Unlike what is seen in CRC, where CD70^+^ CAFs were always found adjacent to the tumor cells, we did not detect such a pattern in PDAC [19, 20]. In CRC, our group also detected CD70^+^ CAFs especially at the invasive front and more abundantly in invasive tumor specimens [19]. We did notice that a high CD70^+^ CAF score was mostly present in samples from PDAC patients with an advanced disease stage, but the lack of adequate PDAC tumor samples from stage T_1_N_x_M_x_ and T_4_N_x_M_x_ is a drawback in this study. More research on a bigger patient cohort is needed to draw firm conclusions on this for PDAC patients. Yet, these data reveal the therapeutical potential of CD70 as a target on both tumor cells and the TME in CRC and PDAC patients.

In the past, it has been demonstrated that targeting CAFs is not without a challenge [14]. For example, elimination of the total CAF population using anti-α-SMA therapy resulted in adverse outcomes in PDAC models [16]. Targeting the fibroblast activation protein proved to be more effective, yet this antigen is also expressed in some healthy tissues like the bone marrow making clinical translation more complicated [17]. Thus, due to the complex and heterogenous nature of CAFs, it is crucial to identify selective targets and develop therapies that can specifically eliminate them. Considering that CD70 is restricted to a subpopulation of CAFs that harbors clear tumor-promoting capacities, selectively targeting CD70^+^ CAFs has the ability to positively modulate the TME in CRC and PDAC patients. Additionally, CD70 is only shortly present on a low number of immune cells, and it has recently been shown in a clinical trial that the number of immune cells was not affected during anti-CD70 treatment of AML patients [45].

We developed a CD70-CAR NK cell therapy to specifically eradicate CD70^+^ tumor cells and CD70^+^ CAFs. In recent years, several CAR T cell therapies targeting CD70^+^ tumor cells have been designed with various antigen-recognition domains (CD27 receptor, single chain variable fragment (scFv), or nanobodies) and intracellular co-stimulatory domains (CD27, CD28 or 4-1BB) [46–54], and some even entered clinical trials mostly targeting hematological malignancies or renal cell carcinoma [6]. Only recently, a few research groups started to develop CD70-CAR cell therapy using NK cells [50, 55]. We generated CD70-directed CAR NK cells based on a truncated CD27 receptor fused to a 4-1BB co-stimulatory domain and a CD3ς signaling domain. Since our results demonstrated only minimal efficacy in more complex models, we explored how we could improve CD70-CAR NK cell functionality. Research conducted by our group has already demonstrated that peripheral blood-derived NK cells exhibit increased potency to kill PDAC tumor and CAF cell lines when prior stimulated with IL-15 [34]. In this study, among a panel of proinflammatory cytokines, stimulation with IL-15 proved to be the most competent in augmenting CD70-CAR NK cell target cell lysis. Furthermore, adding an IL-15 cytokine cassette to the mRNA CAR construct drastically improved CD70^+^ tumor and CAF cell killing in both basic and advanced models. Similarly, other groups have sought to improve the activity of CD70-CAR cell therapy. Indeed, adjusting the structure of the CAR to improve functionality [48, 49, 54], including a second CAR target to prevent relapse due to antigen loss [3], combining the CAR with a PARP inhibitor that positively modulates the TME for CAR T cell infiltration [56, 57] or increasing CD70 target expression (54), knocking-out CD70 to prevent fratricide and exhaustion [51, 52, 58], and including an inhibitory CAR construct to halt trogocytosis-mediated fratricide [59] have also led to improved potency of the CD70-CAR cell product.

Production of fourth-generation CAR constructs or ‘TRUCKs’ (T cells redirected for universal cytokine-medicated killing) expressing cytokines or chemokines is applied broadly in CAR immunotherapy to improve homing, persistence or functional activity of the cell therapy, and to modify the TME and activate bystander immune cells [60]. Rezvani et al. demonstrated that CD19-CAR NK cells armed with IL-15 showed ameliorated persistence and anti-tumor activity in mice [61]. Knocking out the negative feedback loop of IL-15 further enhanced the potency of these IL-15-secreting CD19-CAR NK cells [62]. Additionally, in patients IL-15 stimulation showed improved efficacy of CD19-CAR NK cells by overcoming partly tumor-induced loss of metabolic fitness [63]. Nevertheless, IL-15-associated toxicities due to systemic secretion have been observed [64, 65]. We found that our established CD70-CAR-IL-15 NK cells only secrete very low amounts of IL-15, which mainly have an autocrine/intracellular mode-of-action. Moreover, no IL-15-related toxicities were detected in the in vivo experiment.

Our data imply that the efficacy of CD70-CAR NK cells is dependent on the CD70 antigen load present on the target cells. This was previously also described by Cheng et al. with nanobody-based CD70-targeting CAR T cells in AML, and tackled by promoting CD70 expression on AML cells using epigenetic modulators [52]. Enhancing CD70 expression on target cells to improve efficacy of anti-CD70 therapy has also been achieved with other epigenetic regulators and chemotherapy [27, 45, 66]. Interestingly, we found that increasing the amount of CAR molecules influenced the antigen-dependency of CD70-CAR NK cells. Stimulating CD70-CAR NK cells with IL-15 resulted in a higher CAR density on the cell surface and a boosted activation status, shown by an augmented secretion of IFN-γ and TNF-α, and by an improved balance of activating and inhibitory receptors DNAM-1 and TIGIT, respectively. The former presumably sensitizes CD70-CAR-IL-15 NK cells toward target cells displaying low CD70 antigen expression by increasing CD70/CAR binding.

Exploiting CD70 as anti-cancer target has already shown promising results in clinical trials [6, 67]. In this research, we provide an anti-CD70 cell therapy, CD70-CAR-IL-15 NK cells, with a broad therapeutic window. This cell therapy is capable of eliminating hematological and solid tumor cells with both low and high CD70 expression. Even more, we are the first to show a CD70-directed therapy that can target both the tumor cells and the TME in solid tumors. Furthermore, CD70-CAR-IL-15 NK cells can be produced as a well-defined off-the-shelf cell product. We show high efficiency using CAR mRNA transfection, circumventing the safety hazards of viral transductions. Nevertheless, our promising results would be retained in CD70-CAR-IL-15 NK cells obtained from viral transduction. Along the same lines, considering the beneficial effect of IL-15 on other (CAR) NK cell therapies, we are convinced that our findings are not limited to the NK-92 cell line but can be extrapolated to CAR NK cell therapies derived from primary human NK cell sources.

## Conclusion

In conclusion, we demonstrated that CD70 in CRC and PDAC patients has great therapeutic potential to target both tumor cells and tumor-promoting CAFs. Additionally, we show that armoring CD70-CAR NK cells with IL-15 is a prerequisite for effective eradication of low- and high-expressing CD70^+^ tumor cells and CAFs. Mechanistically IL-15 increased CD70-CAR expression and boosts activation status of CD70-CAR NK cells. Together, these results provide a strong rationale to translate CD70-CAR-IL-15 NK cells to the clinic to improve outcomes for CRC and PDAC patients and potentially other tumor types that are characterized by a strong desmoplastic reaction.

### Supplementary Information

**Additional file 1**. Supplementary figures S1–S5.

## Data Availability

RNA-seq data are available in the TCGA database (PCAWG Firehose). All other data are available in the main text or in the supplementary materials.

## Abbreviations

NK cell: Natural killer cell
PDAC: Pancreatic ductal adenocarcinoma
TME: Tumor microenvironment
CAF: Cancer-associated fibroblast
CRC: Colorectal cancer
CAR: Chimeric antigen receptor
TCGA: The cancer genome atlas
IL-15: Interleukin 15
FFPE: Formalin-fixed paraffin embedded
RSEM: RNA-seq by expectation–maximization
COAD: Colon adenocarcinoma
READ: Rectal adenocarcinoma
PAAD: Pancreatic adenocarcinoma
ICB: Immune checkpoint blockade
H&E: Hematoxylin and Eosin
α-SMA: Alpha smooth muscle actin
mAB: Monoclonal antibody
NSG: NOD-Scid gamma
mRNA: Messenger RNA
IFN-γ: Interferon Gamma
TNF-α: Tumor necrosis factor alpha
ED50: Effector dose 50
CI: Cell index
SD: Standard deviation
SEM: Standard error of mean
MFI: Mean fluorescent intensity
TRUCK: T cells redirected for universal cytokine-medicated killing
PBMC: Peripheral blood mononuclear cell
TIL: Tumor-infiltrating lymphocyte
P/S: Penicillin/streptomycin
DMEM: Dulbecco-modified eagle medium
