# Supplementary material for: IL-15-secreting CAR natural killer cells directed toward the pan-cancer target CD70 eliminate both cancer cells and cancer-associated fibroblasts
Source: J Hematol Oncol. 2024 Feb 9;17:8. doi: 10.1186/s13045-024-01525-w (PMC10854128; doi:10.1186/s13045-024-01525-w)
Supplement: Supplementary file 1 — Additional file 1. Supplementary figures S1–S5. [file 13045_2024_1525_MOESM1_ESM.docx]

**Supplementary figure 1. CD70 expression in PDAC patient samples and healthy donors.**

**A** To validate specificity for CD70 in tumor stroma of the anti-CD70 antibody used in this study, a cohort of 9 historical PDAC tissue samples stained for CD70 with a clinically approved antibody that is not commercially available anymore (1) were consulted and analyzed for CD70^+^ CAFs in the TME. CD70^+^ CAFs were found in 3/9 PDAC patients and representative images of CD70^+^ CAFs- and CD70^-^ CAFs-containing samples are shown. **B** Distribution (%) of the amount CD70^+^ CAFs scored in 23 PDAC patients across disease stage. No PDAC patients with disease stage T1 were included in this study. **C** CD70 expression was assessed on circulating CD4^+^ and CD8^+^ T cells, B cells, NK cells and monocytes derived from PBMCs from four healthy donors. Percentage CD70^+^ cells per immune cell subtype are shown as well as representative histogram overlays of the antibody cocktail containing the CD70 antibody (i.e. CD70) and the antibody cocktail minus the CD70 antibody (i.e. FMO CD70).

**Supplementary figure 2. Flow cytometric detection of CD70-CAR expression and CD70^+^ target cell killing.**

**A** Representative flow cytometry plots depicting the gating strategy for the detection of the CD70-CAR 24 h after electroporation without CAR mRNA (MOCK; upper plots) and with CAR mRNA (CD70-CAR; lower plots). Live cells were gated on the 7-AAD^-^ population (middle right plots) and CD70-CAR^+^ cells were determined by staining for CD27-PE (right plots). **B** Histogram overlays representative for the natural CD27 expression on MOCK NK cells over time after electroporation without CAR mRNA compared to the isotype control. **C** Flow cytometry plots showing the gating strategy to detect CD70^+^ Raji cell survival after 4 h co-culture with MOCK NK cells (upper plots) and CD70-CAR NK cells (lower plots). Raji cells were stained with the PKH67 dye in order to separate target cells (Raji) from effector cells (NK cells; left plots). Percentage target cell survival was determined by staining with Annexin V-PE and 7-AAD and gating on the Annexin V^-^ 7-AAD^-^ cell population.

**Supplementary figure 3. Effect of IL-15 stimulation on the functionality of CD70-CAR NK cells.**

**A** Surface CD70-CAR expression over time (24 h, 48 h and 72 h post-electroporation) on CD70-CAR and CD70-CAR-IL-15 NK cells, with the amount of CD27^+^ cells (left) and the intensity of CD27 expression (right; depicted as mean fluorescence intensity minus isotype control, i.e., ΔMFI; n=4). **B** Expression of immune checkpoint molecules DNAM-1, TACTILE, TIGIT, PD-1 and LAG3 24 h post-electroporation on the cell surface of MOCK, CD70-CAR and CD70-CAR-IL-15 NK cells assessed by flow cytometry. Upper row shows intensity of the expression for the different immune checkpoints, depicted as mean fluorescence intensity minus FMO (fluorescence minus one) control (i.e., ΔMFI; n=5). Lower row shows percentage cells positive for the immune checkpoint (n=5). **C** Effect of exogenous IL-15 stimulation on the CD70-CAR expression, CD70-CAR density on the cell surface (depicted as mean fluorescence intensity minus isotype control, i.e., ΔMFI) and LIM2099 target lysis. CD70-CAR NK cells were either stimulated with the ED50 of IL-15 (2.60 ng/mL, i.e. CD70-CAR + IL-15 ED50) or the highest amount secreted by CD70-CAR-IL-15 NK cells (20 pg/mL, i.e. CD70-CAR + IL-15 SN), and compared to CD70-CAR-IL-15 NK cells that have IL-15 incorporated in the CD70-CAR mRNA construct (n=5). Linear mixed models were used to compare means between CD70-CAR expression and immune checkpoint expression. For the latter, Tukey’s multiple comparison post hoc analysis was applied. ns = p>0.05; * = p<0.05, *** = p<0.001; and ****, p < 0.0001.

**Supplementary figure 4. CD70^+^ Raji-bearing mice treated with CD70-targeting CAR NK cells or MOCK control NK cells.**

**A** After generation and before injection, CD70-CAR NK cells, CD70-CAR-IL-15 NK cells and MOCK NK cells were irradiated with a 10 Gy sublethal dose to prevent outgrowth of the NK-92 cell line. Histogram overlays display the CD70-CAR expression on the cell surface before and right after irradiation. **B** Follow up of the body weight of the Raji-bearing mice after treatment with 1.0 x 10^7^ (CAR) NK cells (two times with three days in between, indicated by the red arrows). Error bars represent mean ± standard error of mean.

**Supplementary figure 5. CD70-CAR NK cells expressing IL-15 show increased activity against CD70^+^ CAFs in PDAC derived microtumors** **A-C** Co-cultures of PDAC patient 002 microtumors (P002) with different treatment conditions: untreated, MOCK control NK cells, CD70-CAR NK cells, and CD70-CAR-IL-15 NK cells. **A** Representative brightfield images with the overlay of the red fluorescent signal for the different treatment conditions. **B** Growth rate normalized against timepoint 0h over time for the different treatment conditions. **C** Quantification of 3 different timepoints (12 h, 24 h and 36 h after treatment), comparing the different treatment conditions normalized to the untreated control at that timepoint (n=3). **D-F** Co-cultures of PDAC patient 044 microtumors (P044) with different treatment conditions: untreated, MOCK control NK cells, CD70-CAR NK cells, and CD70-CAR-IL-15 NK cells. **D** Representative brightfield images with the overlay of the red fluorescent signal for the different treatment conditions. **E** Growth rate normalized against timepoint 0h over time for the different treatment conditions. **F** Quantification of 3 different timepoints (12 h, 24 h and 36 h after treatment), comparing the different treatment conditions normalized to the untreated control at that timepoint (n=3). **G** Comparison of CD70^+^ CAF elimination by CD70-CAR NK cells and CD70-CAR-IL-15 NK cells over the three different patient-derived PDAC microtumors by normalizing the normalized growth rate (total red fluorescent area of timepoint (T)x versus T0) against the MOCK control NK cells. Error bars represent mean ± standard error of mean * = p<0.05, ** = p<0.01, *** = p<0.001 and **** = p<0.0001.
